# Chemical Scalp Burn after Hair Coloring—Case Report with Literature Review

**DOI:** 10.3390/jcm13123542

**Published:** 2024-06-17

**Authors:** Natalia Welc, Sandra Ważniewicz, Aleksandra Anioła, Paulina Ratajska, Małgorzata Wilawer, Magdalena Jałowska, Ryszard Żaba

**Affiliations:** Department of Dermatology, Poznan University of Medical Sciences, 61-701 Ponzan, Polandmjalowska@ump.edu.pl (M.J.); rzaba@ump.edu.pl (R.Ż.)

**Keywords:** chemical burn, scalp, scalp burn, hair dyeing, hair coloring

## Abstract

Hair dyeing is widely performed around the world. Chemical and thermal burns can result from the components present in brightening and coloring products, as well as the application process. We present a case of a chemical burn after applying hair dye and review the literature on similar cases, the composition of hair dyes, their mechanism of action, and the process of burns. The patient was a 17-year-old girl, who presented to the Dermatology Clinic with a 13 × 10 cm ulcer on the scalp after hair dyeing in a hairdressing salon. General and local treatment was applied, with particular emphasis on specialized dressings. The ulcer site was replaced by an area of scarring after 11 months of treatment. Based on the presented case and the review of the literature, we conclude that hair dye treatments warrant careful attention for potential scalp complications. The diagnostic and therapeutic approach demands a multidisciplinary effort, with ongoing patient–doctor cooperation throughout the treatment, which may complicate and span several months.

## 1. Introduction

Hair plays an important role in the current perception of human appearance, determining health, beauty, and attractiveness [1]. Although alopecia is not a life-threatening disease, it may contribute to a significant decline in patients’ quality of life and an increased need for psychological support [2,3,4]. One type of alopecia is scarring alopecia, which, through scarring, leads to irreversible damage to hair follicles and sebaceous glands. This type of alopecia is observed in diseases such as lichen planopilaris, frontal fibrosing alopecia, and folliculitis decalvans, but also after severe chemical and thermal burns [5,6].

One of the procedures that may lead to chemical and/or thermal burns of the scalp is hair dyeing, which is currently commonly performed in hairdressing salons [6]. During the hair lightening treatment, the hair is placed in sections on aluminium foil, and then a mixture of the product with hydrogen peroxide and reaction catalysts is applied. A dryer is often additionally used to speed up the dyeing process by increasing the temperature. The dye is washed off with shampoo and water [7].

So far, only a dozen or so cases of severe chemical and/or thermal burns secondary to treatments aimed at changing hair colour have been described in the literature. We present the case of a young woman who suffered a severe chemical burn to the scalp after a hair-lightening procedure and describe the course of the diagnostic and therapeutic process. We also review the literature on burns related to hairdressing procedures.

## 2. Case Report

A 17-year-old patient was admitted to the Department of Dermatology with an ulcer after a chemical burn on the parieto-occipital part of the scalp. According to the patient, the lesion appeared right after a hairdressing visit, which she attended intending to lighten her hair colour. A moment after applying the preparation to her hair, the woman felt a strong burning sensation on her scalp, after which the preparation was washed off and further activities were discontinued. Initially, the patient experienced erythema, swelling, and painful inflammatory infiltration. The patient was consulted by a dermatologist, who suspected allergic contact dermatitis and implemented bilastine and local treatment with clobetasol propionate. Due to increased pain, increasing swelling, and emerging erosions, the patient was consulted again after 2 days at another dermatology clinic, where zoonotic mycosis of the scalp was considered (due to the presence of a dog in the hairdressing salon during the visit) and terbinafine therapy was started. Over the next 2 weeks, purulent contents appeared from the skin lesions. On the day of admission to the Dermatology Clinic, the patient denied pain. She had no chronic diseases and she was taking rupatadine twice a week due to urticaria.

Clinical examination revealed a deep ulcer measuring 13 × 10 cm with a bottom covered with necrotic masses and purulent contents, with a necrotic eschar in the central area. During hospitalization, general and local treatment was initiated. Laboratory tests showed no clinically significant abnormalities and the inflammatory parameters were negative. A computed tomography of the head was performed to assess the extent of the ulcer, the depth of which was estimated at approximately 5 mm and the diameter at 75 mm. The examination showed no signs of bone destruction or periosteal reactions. Patch tests were also performed and the results were negative.

After surgical consultation, the patient was offered debridement of the necrotic eschar and the wound. A skin graft was also considered. The patient did not consent to the proposed surgical treatment. During hospitalization, therapy was continued with terbinafine at a dose of 250 mg (one tablet once a day) for 10 days, bilastine at a dose of 20 mg (two tablets twice a day), and treatment with ceftriaxone at a dose of 2 g intravenously (once a day) for 7 days. Ointment with betamethasone and gentamicin was used topically.

The patient was discharged with a diagnosis of a chemical burn (corrosive irritant contact dermatitis) in good general condition with improvement. It was recommended to continue treatment with terbinafine 250 mg (once a day) for 3 weeks and ointments with betamethasone and gentamicin, as well as further care at the dermatology clinic.

After obtaining a negative mycological test result, terbinafine was discontinued. Treatment included topical use of silver sulfathiazole (cream) for 5 weeks with a significant improvement in the dermatological condition, then hydrocolloid dressings were used (with active hydrocolloid-carboxymethylcellulose (20%), gelatin (20%), and pectin (20%), suspended in a mass of hydrophobic sodium polyisobutylene (40%)) with significant deterioration of the dermatological condition and the appearance of new erosions. The dressings were then replaced with a hydrocolloid dressing with silver. Due to a lack of improvement, increasing erosions and leakage of purulent content, gauze dressings soaked in paraffin with the addition of chlorhexidine were introduced. A preparation containing 40 ppm (0.004%) HOCl or octanidine was used to wash the wound, resulting in a slow improvement in the dermatological condition. A plastic surgeon consulted the patient twice—but a skin graft was not performed. Currently, the patient is waiting for surgical treatment, it is planned to install an expander and remove the scarring area. The patient was referred for hyperbaric oxygen therapy, however, she was disqualified from the treatment because of pectus excavatum. The therapy was continued for 11 months on an outpatient basis until the burn healed and a scar formed. Throughout the entire treatment period, the patient did not attend school and received individual education. The patient was advised to refrain from further hair coloring procedures.

**Figure 1:**
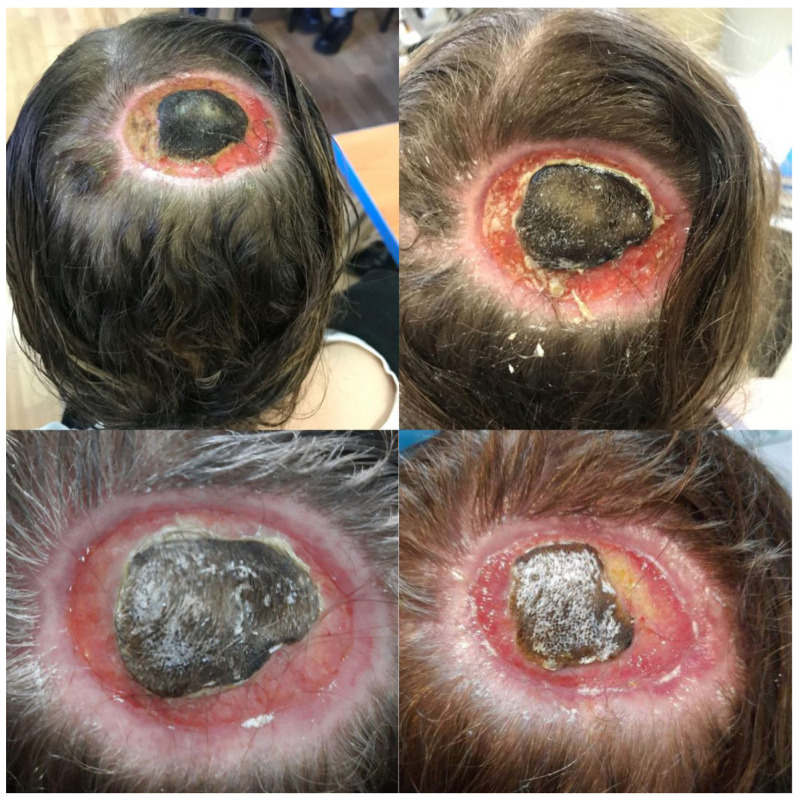
Appearance of the lesion during treatment-ulceration after chemical burn in the parietal-occipital part of the scalp. A necrotic eschar is visible in the central part, the surrounding skin is healing.

**Figure 2:**
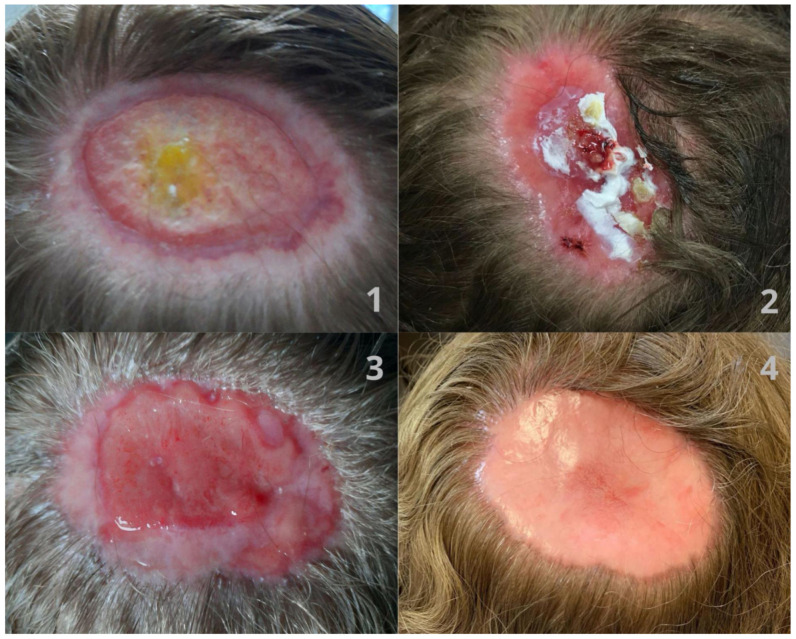
The appearance of the lesion during treatment-ulcer after chemical burn in the parieto-occipital part of the scalp (**1**–**3**) during the healing process, complicated by the enlargement of erosions and leakage of purulent contents (**2**). After complete healing, an area of scarring is visible at the site of the original ulcer (**4**).

The course of the 11-month treatment was documented photographically with the patient’s consent (Figure 1 and Figure 2).

Six months after complete healing of the wound, the patient eventually agreed to the plastic surgery procedure. An expander was inserted in the upper part of the scar to stretch the skin, excise the scar area, and suture the scalp back together afterwards. Unfortunately, a week after implanting the expander, a purulent discharge appeared and the expander must have been taken out. The wound healed without any further complications. The patient consented to another tissue expander implantation after 6 months and will stay under treatment at the Dermatology Clinic.

## 3. Discussion

Every third woman over 18 years of age and every tenth man over 40 years of age in Europe and the United States dyes their hair [8]. Hair dyes include temporary, semi-permanent, and permanent dyes. The effect depends on the depth of penetration into the hair shaft and the time of exposure to the dye ingredients. Moreover, depending on the mechanism of action, oxidative and non-oxidative dyes are distinguished [9]. Permanent hair dyes consist of three main groups of ingredients. Precursors include aromatic amines, e.g., p-phenylenediamine (PPD), toluene-2,4-diamine (PTD), and p-aminophenol. Among the coupling compounds are m-phenylenediamines, resorcinol, and naphthol [10]. Oxidising compounds are hydrogen peroxide (H_2_O_2_), potassium, and ammonium persulfate [11]. The standard concentration of hydrogen peroxide in brightening dyes is 3–6%.

A particularly high risk of chemical burns on the scalp occurs when using preparations with H_2_O_2_ concentrations higher than 10$, although the literature describes cases of severe chemical burns also at lower concentrations, which are generally considered safe [12,13]. In this particular case, we suspect that it was the excessively high concentration of H_2_O_2_ in the hair dye that was the cause of the chemical burn. The scalp is characterized by an increased ability to absorb chemicals due to the large number of hair follicles, the composition of sebum and the superficial location of capillaries [14]. Mild and severe burns are distinguished depending on the degree of damage to the scalp. In the case of a mild burn, erythema and blisters occur. A symptom of a severe burn is the development of inflammation. Direct contact of oxidizing substances with the skin causes coagulative necrosis. Due to overheated aluminium foils and hair dryers, thermal burns may coincide [9].

So far, several cases of chemical and/or thermal burns of the scalp caused by the use of hair dyes have been described in the literature. The reports concerned patients aged 12–30. The first symptoms reported by patients included strong, throbbing pain, tingling and burning sensations, an unpleasant feeling of heat, and erythema, which occurred during or immediately after the procedure. In the case of patients who additionally used a helmet dryer, the first symptoms appeared after 2–5 min. In all of the described patients, scalp ulceration occurred within 10–21 days after the procedure [13].

It is important to underline that such incidents as reported here are very rare. Moreover, it is worth mentioning that after analyzing the available literature, it can be concluded that the case we describe presents a patient with the most extensive ulcer (13 × 10 cm) after a chemical burn with hair dye.

The differential diagnosis should include bacterial and fungal skin infections and pemphigus, as well as other autoimmune blistering diseases, squamous cell carcinoma of the skin, and dermatitis artefacta [15,16,17]. Treatment of chemical burns on the scalp includes the use of local glucocorticosteroids and disinfectants [15,18]. Antibiotic therapy is recommended only in the case of bacterial superinfection of the wound [18]. Skin grafts are used in surgical treatment [12]. Treatment may be complemented by hair follicle transplantation [19].

A serious complication of scalp burns is irreversible damage to the hair follicles, which results in cicatricial alopecia [9]. Moreover, carcinogenesis and the development of squamous cell carcinoma of the skin may occur within the scar [20].

Another phenomenon observed in people undergoing hair dyeing treatments is allergic reactions [10]. Symptoms of contact allergy, such as redness, burning, and itching of the skin may occur up to 24 h after the treatment [1]. It is also possible to develop allergic contact dermatitis, which affects the skin of the scalp, face, neck, and hands. Patients then present with redness of the skin with the presence of blisters and subsequent peeling of the skin [10].

The influence of chemical compounds contained in hair dyes on carcinogenesis was also analyzed. The research focused particularly on the risk of developing bladder cancer, breast cancer, and haematological cancers. The results of meta-analyses are inconsistent, which makes it impossible to draw clear conclusions. Due to existing reports on the increased risk of cancer development in people who use hair dyes, it is necessary to continue research in this direction [10,21,22].

## 4. Conclusions

This case highlights the potentially serious complications associated with the widely practised hair dyeing procedure in hair salons worldwide. Notably, hair coloring is also common among adolescents, indicating that the issue may extend to the pediatric population. It is crucial to underscore that, beyond the cosmetic concerns, such patients may also endure lasting damage to their mental well-being. Therefore, it is imperative to educate those opting for hair dyeing on the potential risks, including chemical and/or thermal burns. Strict adherence to the guidelines for coloring products and equipment is essential, and each procedure should be executed meticulously to mitigate associated hazards.

## Data Availability

The data presented in this study are available on request from the corresponding author.
